# Experimental Comparison and Empirical Path Loss Modeling of LoRa Communication in Line-of-Sight and Forest Environments at 923 MHz

**DOI:** 10.3390/s26103192

**Published:** 2026-05-18

**Authors:** Kamol Boonlom, Jarun Khonrang, Prayoot Akkaraekthalin, Nonchanutt Chudpooti

**Affiliations:** 1 Faculty of Industrial Technology, Chiang Rai Rajabhat University, Chiang Rai 57100, Thailand; kamol.boo@crru.ac.th (K.B.); jarun.kho@crru.ac.th (J.K.); 2 Department of Electrical and Computer Engineering, Faculty of Engineering, King Mongkut’s University of Technology North Bangkok, Bangkok 10800, Thailand; prayoot.a@eng.kmutnb.ac.th; 3 Department of Industrial Physics and Medical Instrumentation, Faculty of Applied Science, King Mongkut’s University of Technology North Bangkok, Bangkok 10800, Thailand

**Keywords:** LoRa, path loss modeling, received signal strength (RSSI), wireless sensor networks, forest propagation

## Abstract

This study presents a measurement-driven comparison of LoRa communication performance in two tropical deployment scenarios at 923.2 MHz: an open line-of-sight (LOS) path and a forest-obstructed path. To ensure a controlled comparison, both scenarios were evaluated over the same transmission distance of 1.2 km using identical radio configuration, antenna heights, and hardware settings. Field measurements were conducted from 50 m to 1.2 km in 50 m increments, with three repeated measurements at each distance point. The measured RSSI decreased from −60.52 dBm to −89.48 dBm in the LOS case and from −77.62 dBm to −114.62 dBm in the forested case. Using a bandwidth of 125 kHz and a receiver noise figure of 6 dB, the corresponding estimated SNR at 1.2 km was 27.55 dB for the LOS path and 2.41 dB for the forested path. Relative to the free-space baseline, the measured LOS link showed a deviation of 31.14 dB at 1.2 km, while the forested link showed a deviation of 56.28 dB. The additional attenuation specifically associated with the forested environment was approximately 25.14 dB, with a mean excess loss of 24.70 dB over the full route. Regression analysis further yielded effective path-loss exponents of 2.31 for the LOS case and 3.22 for the forested case. Based on these results, a site-specific empirical correction approach and an approximate 25 dB first-order design margin are suggested for preliminary LoRa link-budget planning in similar tropical vegetated environments. The findings indicate that free-space-only prediction may be insufficient for practical deployment and that measurement-driven correction can improve the realism of wireless sensor network design in vegetation-rich environments.

## 1. Introduction

Low-power wide-area networks (LPWANs) have become a key enabler of large-scale Internet of Things (IoT) systems [1,2] that require long-range connectivity with low energy consumption [3,4]. Unlike short-range wireless technologies that prioritize high data rates [5], LPWANs are designed to support battery-powered sensing nodes distributed across wide geographical areas, particularly in rural and harsh outdoor environments [6,7,8]. Among these technologies, LoRa has gained widespread adoption due to its long transmission range, adaptive spreading factor capability, and operation in unlicensed sub-GHz frequency bands [9,10,11,12]. These features make LoRa particularly suitable for environmental monitoring [13], smart agriculture [14], and infrastructure sensing applications [15], where low deployment cost and long-term reliability are essential.

In environmental sensing applications, such as river level monitoring and flood warning systems, sensor nodes are typically deployed in remote areas without access to stable power infrastructure. These nodes often rely on battery or solar energy sources and must maintain reliable communication over long distances to ensure continuous data acquisition. In tropical regions such as Thailand, deployment environments frequently include dense vegetation, mixed terrain, and varying canopy structures. These environmental factors significantly influence radio wave propagation, leading to deviations from ideal propagation conditions assumed during initial system design.

Although LoRa systems can achieve multi-kilometer communication under ideal line-of-sight (LOS) conditions, practical deployments rarely satisfy free-space assumptions. Signal propagation in vegetated environments is affected by absorption, scattering, and diffraction mechanisms [16,17,18], particularly when the first Fresnel zone is partially obstructed [19,20]. As a result, the actual received signal strength may differ substantially from predictions based on conventional free-space path loss (FSPL) models. While empirical and semi-empirical models have been proposed to account for vegetation effects, their accuracy is highly dependent on deployment-specific parameters such as forest density, antenna height, and propagation geometry. Furthermore, many existing studies employ heterogeneous measurement conditions or varying link distances, making it difficult to isolate and quantify the true impact of environmental factors on LoRa communication performance.

In addition to propagation loss, LoRa communication performance is influenced by modulation characteristics and receiver sensitivity. Due to its chirp spread spectrum modulation, LoRa can successfully demodulate signals even at negative signal-to-noise ratio (SNR) levels depending on the selected spreading factor. However, in obstructed environments where additional attenuation and fading occur, the available link margin becomes limited, and communication reliability becomes sensitive to environmental variations such as foliage movement, multipath propagation, and partial Fresnel zone obstruction. Therefore, establishing a clear relationship between propagation conditions and measurable performance metrics, including received signal strength indicator (RSSI), SNR, and link margin, is essential for practical system design. Measurement-driven characterization approaches have also been applied in related electromagnetic and sensing systems, demonstrating the importance of experimental validation in complex environments [21,22]

To address these challenges, this study presents a measurement-driven comparative analysis of LoRa communication performance in two representative tropical deployment scenarios: an open line-of-sight (LOS) environment and a forest-obstructed environment. In contrast to prior work, a controlled experimental framework is established by fixing the transmission distance at 1.2 km for both scenarios, enabling a direct and consistent comparison of environmental effects. Field measurements are conducted using a LoRa system operating at 923.2 MHz, where key performance metrics, including RSSI and SNR, are systematically evaluated under identical system configurations.

Based on the experimental results, this study quantifies the excess attenuation introduced by forests and examines the impact of partial Fresnel zone obstruction on signal degradation. Rather than treating the discrepancy between model prediction and measurement as a simple error, this work interprets it as a practical design parameter that can be incorporated into link budget planning. The results are further used to derive deployment-oriented insights, including link margin considerations and configuration guidelines for transmit power and spreading factor selection.

The main contributions of this study are threefold. First, a controlled field comparison of LoRa propagation in open and forested environments at an identical transmission distance is presented, enabling a clear evaluation of environmental effects. Second, the discrepancy between free-space-based predictions and measured RSSI and SNR is quantitatively analyzed and interpreted using Fresnel zone considerations. Third, practical design insights are derived from measurement data to support reliable and energy-efficient deployment of IoT-based environmental sensing systems in tropical environments.

It should be noted that propagation behavior in urban environments differs significantly from that in rural and forested scenarios. In urban areas, signal propagation is strongly influenced by building-induced blockage, multipath reflections, and street canyon effects, which lead to different attenuation and variability characteristics compared to vegetation-dominated environments. The focus of this study is on rural and forested deployments, which are particularly relevant for environmental monitoring applications. A comprehensive investigation of urban propagation is therefore considered beyond the scope of this work and is identified as an important direction for future research.

## 2. Propagation Modeling and Link Budget Analysis

Designing reliable LoRa links for rural IoT monitoring requires propagation modeling that remains valid across heterogeneous terrain. In practical deployments, a single link may traverse open fields, partially obstructed forest corridors, and vegetation-dense regions, causing received signal levels to deviate substantially from ideal free-space predictions. To address this issue, this study adopts a comparative modeling framework that (i) establishes a baseline prediction using free-space path loss, (ii) characterizes large-scale propagation behavior using the log-distance model and extracted path-loss parameters, (iii) incorporates forest-induced excess loss using an empirical attenuation representation, and (iv) evaluates diffraction effects using Fresnel zone clearance. These analytical components are used to interpret field-measured RSSI and SNR, quantify model–measurement discrepancies, and support deployment-oriented link design.

**Figure 1:**
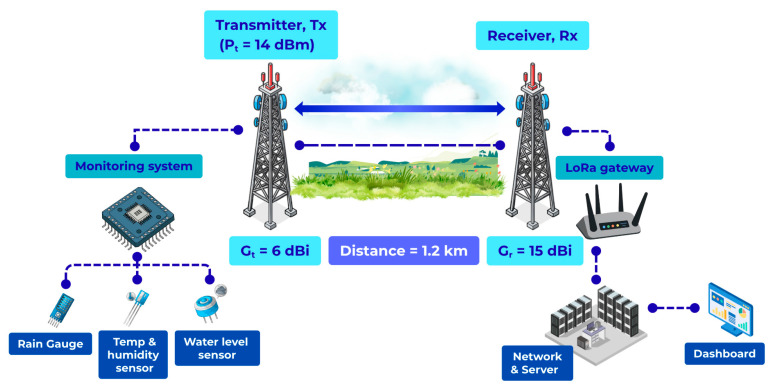
The Line-of-Sight (LOS) free-space path loss diagram.

**Figure 2:**
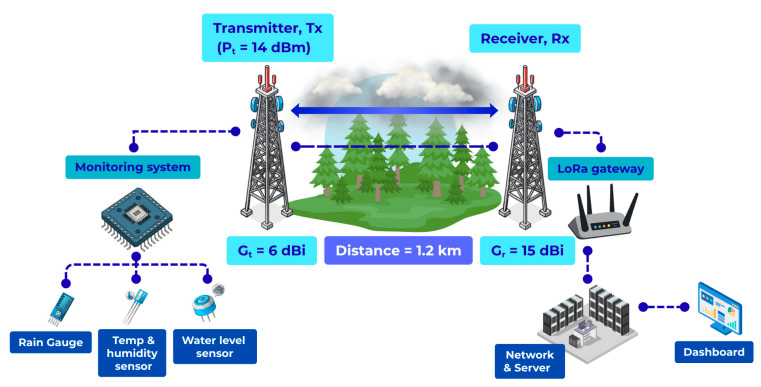
The forest environment path loss diagram.

Two representative propagation scenarios are considered, both operating at 923.2 MHz. The first scenario is an open line-of-sight (LOS) link of 1.2 km with near-complete Fresnel zone clearance, as illustrated in Figure 1. The second scenario is a forested/vegetated link of the same distance with partial Fresnel zone obstruction, as illustrated in Figure 2. These scenarios are selected to enable a direct comparison between ideal and vegetation-obstructed propagation conditions under identical transmission distance, frequency band, and radio configuration.

The LOS scenario represents a baseline propagation condition in which attenuation is primarily governed by distance-dependent spreading loss with minimal environmental obstruction. In contrast, the forested scenario introduces additional attenuation mechanisms, including absorption by foliage, scattering from irregular canopy structures, and diffraction due to incomplete Fresnel zone clearance. These mechanisms result in both increased path loss and greater signal variability.

This controlled configuration enables a direct comparison of propagation behavior in open and forest-dominated environments under identical conditions. Unlike conventional studies that rely solely on theoretical models or simulation-based predictions, this work emphasizes a measurement-driven validation framework in which model predictions are directly compared against field-observed RSSI and SNR. This approach enables the identification of practical discrepancies and supports the development of deployment-oriented link design guidelines.

### 2.1. Link Budget Representation and Measurable Metrics

All link budget quantities in this study are expressed in decibel units (dB/dBm) unless otherwise stated. The received power, $P_{r}$, for a LoRa point-to-point link can be expressed using a standard link budget formulation [23]:

(1) $$\begin{array}{c} P_{r}(d)=P_{t}+G_{t}+G_{r}-PL(d)-L_{s} \end{array}$$

where $P_{t}\;$ is the transmit power (dBm), $G_{t}\;$ and $G_{r}$ are the transmit and receive antenna gains (dBi), $PL\left(d\right)$ is the propagation path loss (dB) at distance, d, and $L_{s}\;$ represents additional system losses (dB) due to cables, connectors, mismatch, and installation-related losses.

In practice, LoRa receivers typically report RSSI and SNR. While RSSI may differ from the absolute calibrated received power due to chipset-dependent offsets, it provides a consistent metric for comparative propagation analysis [24] when the same hardware and configuration are used across measurements. Therefore, this study validates model trends primarily through comparisons between predicted and measured RSSI/SNR behavior in the two environments.

SNR is related to received signal power and the effective noise level [25]:

(2) $$\begin{array}{c} SNR=P_{r}-N \end{array}$$

where N represents the receiver noise level (dBm). Because LoRa demodulation can succeed under negative SNR depending on the spreading factor (SF), link robustness is evaluated using the link margin M, defined as:

(3) $$\begin{array}{c} M=P_{r}-P_{sens} \end{array}$$

where $P_{sens}$ is the receiver sensitivity corresponding to the selected LoRa configuration (SF, bandwidth, coding rate). A positive margin indicates that the link can tolerate additional fading, shadowing, or environmental variability.

### 2.2. Free-Space Baseline Modeling for the Open LOS Scenario

The free-space path loss (FSPL) model provides a theoretical lower bound on attenuation under unobstructed LOS conditions and is used here as a baseline reference for the open 1.2 km scenario:

(4) $$\begin{array}{cc} PL_{FS}(d)=20{log}_{10}(d)+20{log}_{10}(f)+32.44 & \end{array}$$

where ${PL}_{FS}\left(d\right)$ is free-space path loss (dB) at distance d,with d expressed is in kilometers and f is in MHz. At 923 MHz, (4) provides a convenient reference for initial link feasibility assessment and for defining “excess loss” in real deployments.

In this study, FSPL is not treated as an accurate predictor of real-world performance but rather as a reference baseline against which environment-induced excess loss is quantified. However, practical rural links rarely behave as ideal free space due to near-ground antenna placement, local terrain undulation, and multipath from ground reflection. For this reason, FSPL is not treated as the “true” propagation model; instead, it is used as a baseline. The difference between FSPL prediction and measured RSSI/received power is interpreted as an environment- and installation-dependent excess loss, which is later incorporated into margin planning and configuration guidance.

### 2.3. Log-Distance Model and Path Loss Parameter Extraction

To characterize large-scale propagation beyond free-space assumptions, this study adopts the log-distance path loss model:

(5) $$\begin{array}{c} PL(d)=PL(d_{0})+10n{log}_{10}\left(\frac{d}{d_{0}}\right)+X_{\sigma } \end{array}$$

where $d_{0}\;$ is a reference distance, n  is the path loss exponent, and $X_{\sigma }\;$ is a zero-mean Gaussian random variable representing large-scale shadowing with standard deviation σ.

The parameters n and σ provide a compact and deployment-relevant description of propagation behavior. In this study, these parameters are extracted by computing a measurement-based path loss estimate from RSSI (or received power) using the link budget relationship in (1). Specifically, assuming RSSI is proportional to received power under fixed hardware settings, a measured path loss can be approximated as:

(6) $$\begin{array}{cc} PL_{meas}(d)\approx P_{t}+G_{t}+G_{r}-{RSSI}_{LOS}(d)-L_{s} & \end{array}$$

A linear regression of $PL_{meas}\left(d\right)\;$ versus ${log}_{10}(d)\;$ is then used to estimate the path loss exponent n. This step is important for two reasons:

(i) it quantifies how strongly the environment deviates from free-space behavior, and
(ii) it provides a more realistic propagation model for planning and optimization than FSPL alone.

### 2.4. Modelling Excess Attenuation in Forested/Vegetated Terrain

For the 1.2 km forested scenario, additional attenuation is introduced by absorption in foliage, scattering from branches, and diffraction due to partial obstruction. A practical approach used in this work is to represent total loss as FSPL plus an excess attenuation term:

(7) $$\begin{array}{cc} PL_{forest}(d)=PL_{FS}(d)+A_{f}(d) & \end{array}$$

where $A_{f}(d)$ is the forest-induced excess loss. A commonly used deployment-oriented representation assumes excess loss increases with the effective distance within forest:

(8) $$\begin{array}{cc} A_{f}(d)=\alpha d_{f} & \end{array}$$

where $d_{f}\;$ is the effective path length through forest (m) and α  (dB/m) is an empirical attenuation coefficient reflecting foliage density, canopy structure, and seasonal conditions [26,27].

In this work, (7) and (8) are used as an engineering prediction tool rather than a microscopic scattering model. The key objective is to quantify how much additional attenuation is observed in a tropical vegetated environment relative to the open LOS baseline at the same frequency band and radio configuration. Because forest loss can fluctuate due to spatial variation, leaf moisture, and canopy thickness, the comparison is performed using averaged measurement samples, and the remaining discrepancy is treated as a practical uncertainty margin for link design. In addition, the excess attenuation term $A_{f}\left(d\right)\;$ will be estimated from field measurements in the subsequent section, enabling the derivation of a practical empirical correction model for LoRa deployment in tropical vegetated environments. Propagation in vegetated environments has been widely studied, with models such as ITU-R P.833 providing empirical formulations for vegetation attenuation [28]. Recent studies have also investigated LoRa propagation under forest conditions, highlighting the impact of foliage density and environmental variability [29,30]. These models provide useful references; however, their applicability remains dependent on deployment-specific conditions, motivating the measurement-driven approach adopted in this work.

### 2.5. Fresnel Zone Clearance as a Link-Quality Indicator

Even when LOS exists geometrically, partial obstruction of the first Fresnel zone can introduce substantial diffraction loss and increased fading variability. The first Fresnel zone radius is expressed as [20]:

(9) $$\begin{array}{cc} r_{1}=\sqrt{\frac{\lambda d_{1}d_{2}}{d_{1}+d_{2}}} & \end{array}$$

where λ is the wavelength and $d_{1}$, $d_{2}\;$ are the distances from the obstruction point to the transmitter and receiver, respectively.

For link planning, a widely used guideline is that at least 60% of the first Fresnel zone should be clear to avoid significant diffraction-related degradation. In the forested scenario considered in this study, the Fresnel zone clearance is substantially reduced due to canopy obstruction along the propagation path.

The clearance is estimated based on the theoretical first Fresnel zone radius, which depends on the operating frequency, transmission distance, and the relative geometry between the transmitter and receiver. Using the system parameters at 923.2 MHz and a link distance of 1.2 km, the Fresnel zone radius is calculated and compared with the effective obstruction height introduced by the forested environment, including vegetation-induced and terrain-related effects. Based on this geometric analysis, the Fresnel zone clearance is estimated to be approximately 37.05%, which is significantly below the commonly recommended threshold.

This reduced clearance indicates that a substantial portion of the Fresnel zone is obstructed, leading to additional diffraction loss along the propagation path. As a result, the observed attenuation in the forested link cannot be attributed solely to foliage absorption and scattering, but must also account for diffraction effects caused by incomplete Fresnel zone clearance. This provides a physically meaningful explanation for why the measured signal attenuation exceeds the prediction of the free-space path loss (FSPL) model.

### 2.6. Comparative Model Validation Strategy

A key contribution of this work is not only to apply propagation models, but to evaluate how well these models align with field measurements in two representative tropical rural environments. Model validation is performed by comparing predicted received power trends (via the link budget) against measured RSSI/SNR behavior for both scenarios.

To quantify prediction accuracy, the deviation between modeled and measured received signal levels is defined as:

(10) $$\begin{array}{cc} \Delta (d)=P_{r,meas}(d)-P_{r,model}(d) & \end{array}$$

where $P_{r,model}\left(d\right)\;$ is obtained from (1) using either $PL_{FS}\left(d\right)\;$ or $PL_{forest}(d)$, depending on the scenario. The deviation $\Delta \left(d\right)\;$ is interpreted as a combination of (i) unmodeled environmental losses, (ii) installation-related losses, and (iii) large-scale fading variability. In the subsequent results section, $\Delta \left(d\right)\;$ is used to quantify model–measurement discrepancy and to motivate the practical link margin required for robust deployment. This deviation is not treated merely as modeling error but is interpreted as a practical uncertainty term that should be incorporated into link margin design for reliable real-world deployment.

## 3. Experimental Setup and Measurement Methodology

This section describes the deployment configuration, radio settings, baseline analytical estimation, and measurement procedure used to evaluate propagation behavior in open and forested environments. The objective is to ensure reproducibility and to establish a consistent experimental framework for validating the propagation models presented in Section 2.

The experimental setup and measurement methodology provide the basis for the results presented in the following section, where the measured RSSI and SNR are analyzed in relation to the propagation models. The measurements were conducted in a typical tropical environment characterized by medium-density forest and mixed terrain conditions, representative of practical outdoor deployment scenarios.

### 3.1. Deployment Scenarios and Geometry

Field experiments were conducted in a rural tropical environment to evaluate LoRa propagation performance under realistic outdoor conditions. Two distinct propagation scenarios were considered at the same operating frequency (923.2 MHz) and identical transmission distance of 1.2 km, enabling a direct and controlled comparison of environmental effects on signal propagation.

The first scenario corresponds to an open line-of-sight (LOS) link deployed in an unobstructed rural area with minimal forest between the transmitter and the gateway. The second scenario represents a forested or vegetated link at the same distance, where the propagation path is partially obstructed by tree canopy, resulting in reduced Fresnel zone clearance and increased attenuation.

The transmitter-side antenna was mounted at a height of approximately 1.7 m, while the gateway antenna was installed at a height of approximately 8 m above ground level. In the forested scenario, surrounding trees have typical heights between 5–8 m, leading to partial obstruction of the first Fresnel zone. Based on the geometric analysis presented in Section 2, the Fresnel zone clearance along the 1.2 km forested link is estimated to be approximately 37.05%, indicating that diffraction effects are expected to contribute significantly to additional attenuation.

The communication nodes used in this study are configured as LoRa transmission and reception units for propagation measurement purposes only. The sensing functionality of the node is not considered in this work, and the system is used solely as a communication platform for evaluating RSSI and SNR under different propagation environments.

**Figure 3:**
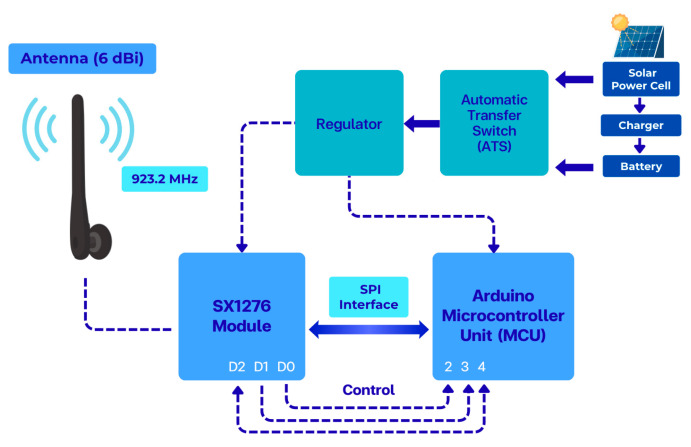
Block diagram of the LoRa communication end node used for RSSI and SNR measurements, including control, power, and wireless transmission subsystems.

**Figure 4:**
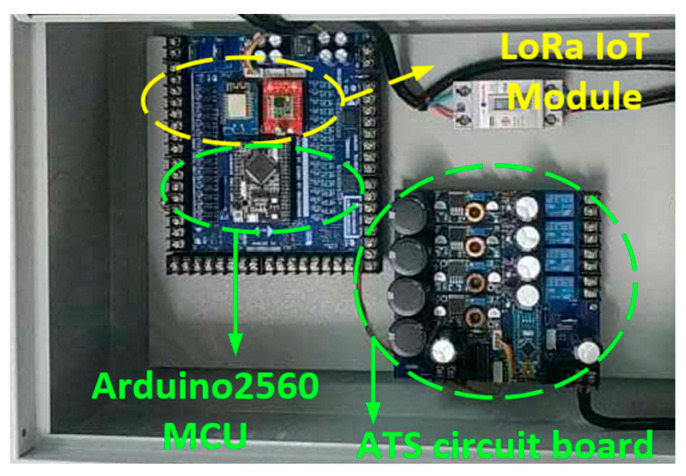
Hardware prototype of the LoRa communication end node used in the field measurements.

The structure of the LoRa communication end node used in the measurement system is illustrated in Figure 3, showing the main functional blocks for signal transmission, control, and power supply. The corresponding hardware implementation of the communication node used in the field measurements is shown in Figure 4.

### 3.2. LoRa Radio Configuration

All experiments were conducted using identical LoRa physical-layer settings to ensure that any observed differences in received signal strength indicator (RSSI) and signal-to-noise ratio (SNR) are attributable solely to propagation conditions rather than configuration variations. The radio parameters used in this study are summarized in Table 1.

The LoRa communication system is based on the SX1276 chipset, which supports sub-GHz operation and enables reliable communication under low signal-to-noise ratio conditions. The system operates at a carrier frequency of 923.2 MHz with a bandwidth of 125 kHz and a spreading factor of SF10, providing a balance between communication range and data rate.

The operating frequency of 923.2 MHz corresponds to the ISM band used for LoRa communication in Thailand. While slight differences exist across sub-GHz bands (e.g., 868 MHz, 915 MHz), the propagation characteristics and observed trends are expected to remain comparable, allowing the results to be used as a first-order reference for similar frequency bands.

The transmitted power was configured at the maximum allowable level and verified using a signal analyzer to ensure consistency with the nominal setting. The measured maximum output power was approximately 14 dBm, confirming alignment with the configured transmission level. The transmitted power reproducibility observed during repeated verification measurements was within approximately ±0.1 dB. In addition, the typical measurement uncertainty of the signal analyzer is approximately ±0.5 dB according to the manufacturer specification. While minor variations may occur in practical operation, no significant fluctuations were observed during the measurement campaign.

Both transmitter and receiver employed omnidirectional antennas, and no changes were made to antenna configuration, placement, or orientation between the two propagation scenarios. This consistency ensures that any variations in RSSI and SNR are directly related to environmental propagation effects, including forest-induced attenuation and Fresnel zone obstruction, rather than differences in hardware or system configuration.

It should be noted that the communication system is configured specifically for propagation measurement, and the transmitted data packets are used only to enable RSSI and SNR evaluation. No application-layer data or sensing information is considered in this study.

### 3.3. Baseline Link Budget Estimation

Prior to field measurements, a baseline received signal level was estimated using the link budget formulation described in Section 2. The free-space path loss (FSPL) model was adopted as a reference prediction for the open line-of-sight (LOS) scenario.

For completeness, a small atmospheric attenuation term of approximately 0.003 dB/km was included in the calculation. However, this contribution is negligible compared to distance-dependent propagation loss and environment-induced attenuation. Under the configured system parameters, the baseline predicted received signal level for the open LOS scenario at a transmission distance of 1.2 km is approximately −58.34 dBm. This value is derived from the FSPL model and serves as a deterministic reference for evaluating deviations observed in field measurements.

Rather than treating discrepancies between predicted and measured values as modeling errors, this study interprets such differences as environment-dependent excess attenuation. In particular, the baseline prediction enables direct quantification of additional losses introduced by forest, partial Fresnel zone obstruction, and installation-related factors in the forested scenario under identical transmission distance conditions.

The baseline estimation was performed using analytical calculation based on the link budget formulation. The results are used solely as a reference baseline, while the primary analysis and conclusions of this study are derived from field measurements.

**Figure 5:**
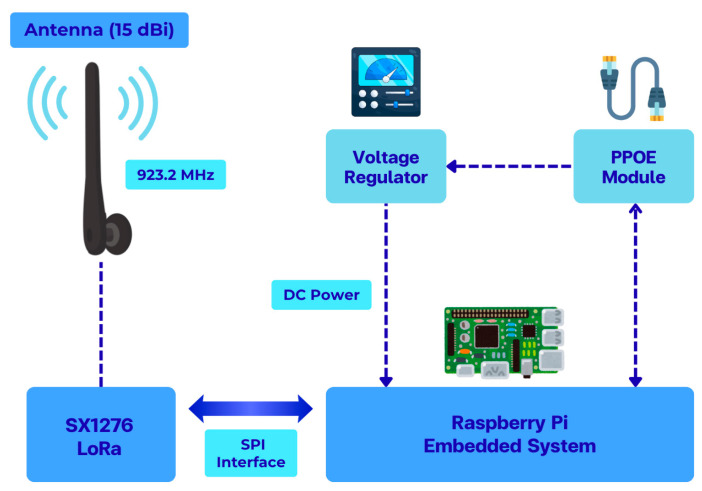
Block diagram of the signal-channel LoRa gateway used for signal reception and backhaul communication.

**Figure 6:**
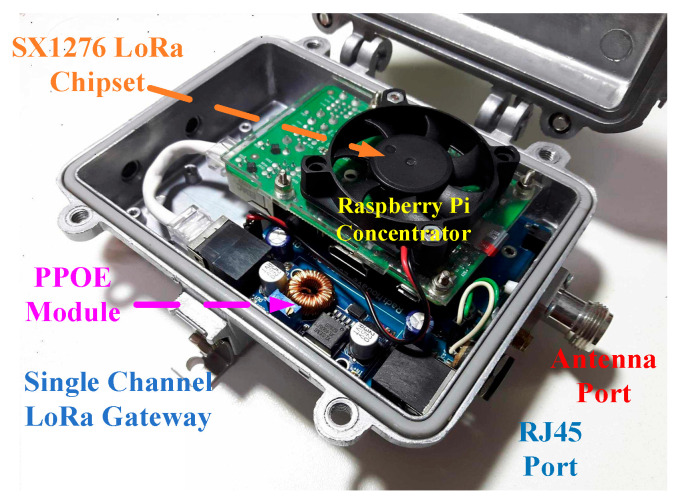
Prototype of the single-channel LoRa gateway used in the field deployment.

The configuration of the LoRa gateway used for signal reception is illustrated in Figure 5, including the signal processing and communication interface. The hardware prototype of the gateway deployed in the field measurement is shown in Figure 6.

### 3.4. Measurement Procedure

Field measurements were conducted by transmitting LoRa packets from the end node to the gateway under the fixed radio configuration described in Table 1. Two measurement procedures were employed in this study.

First, to characterize distance-dependent propagation behavior, measurements were performed at 24 distance points from 50 m to 1.2 km in 50 m increments for both the LOS and forested scenarios. At each distance point, the measurement was repeated three times under identical hardware configuration, antenna orientation, and deployment geometry. It should be noted that the three repeated measurements at each distance point are intended to verify measurement consistency under controlled conditions, rather than to provide statistically exhaustive sampling. The primary objective of the measurement campaign is to capture large-scale propagation trends as a function of distance. The average RSSI and standard deviation were calculated from these repeated measurements.

Second, for fixed-link evaluation at the final deployment distance of 1.2 km, a larger packet set was collected to assess link stability. In this case, 100 transmission samples were recorded for each scenario and used to compute representative mean link-quality values.

The recorded RSSI values were used as practical indicators of received signal strength under identical system conditions. The corresponding SNR was derived from the measured RSSI using the assumed receiver noise floor. The noise floor was calculated based on a bandwidth of 125 kHz and a receiver noise figure of 6 dB, resulting in an effective noise floor of approximately −117.03 dBm. All measurements were conducted within a limited time window and under consistent environmental conditions to minimize temporal variability caused by weather and environmental dynamics. This methodology ensures that the collected data accurately reflects large-scale propagation behavior and provides a reliable basis for comparative analysis and model validation.

**Figure 7:**
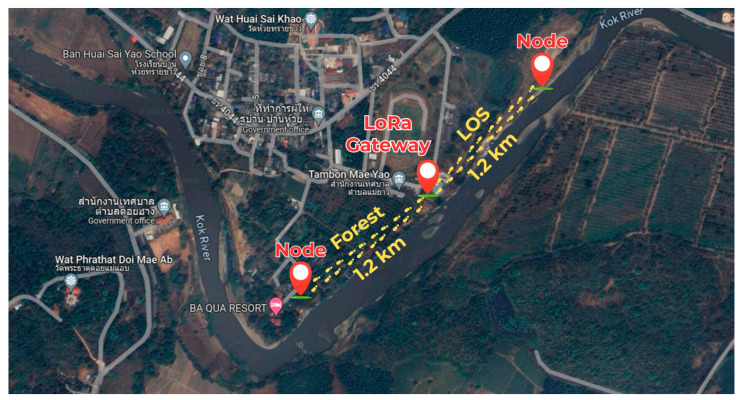
Geographical mapping of the measurement route, indicating the positions of the transmitter (end node), the gateway, and the propagation paths for LOS and forested scenarios. Distance measurement points from 50 m to 1.2 km are illustrated along the route.

To provide a clear spatial representation of the measurement campaign, the geographical mapping of the deployment route is illustrated in Figure 7. The figure shows the positions of the transmitter (end node) and the gateway, along with the propagation paths for both the LOS and forested scenarios. The measurement points are distributed along the route from 50 m to 1.2 km in 50 m increments, enabling direct correlation between the measured RSSI/SNR results and the physical propagation environment.

The measurements were conducted during daytime conditions to ensure consistent visibility, stable operating conditions, and safe deployment in the forested environment. The measurement campaign was carried out over a period from 9 November 2025 to 16 November 2025. Detailed timestamp and GPS coordinates for each measurement point were not recorded, as the focus was on controlled spatial comparison along a fixed route. Future measurement campaigns will incorporate GPS-referenced and time-stamped measurements to enable more comprehensive analysis of temporal, spatial, and environmental variability.

## 4. Experimental Results and Discussion

This section presents the experimental results of RSSI and SNR measurements for both line-of-sight (LOS) and forested propagation scenarios. The results are analyzed in relation to the propagation models introduced in Section 2 and the experimental setup described in Section 3.

### 4.1. RSSI Performance as a Function of Distance

**Figure 8:**
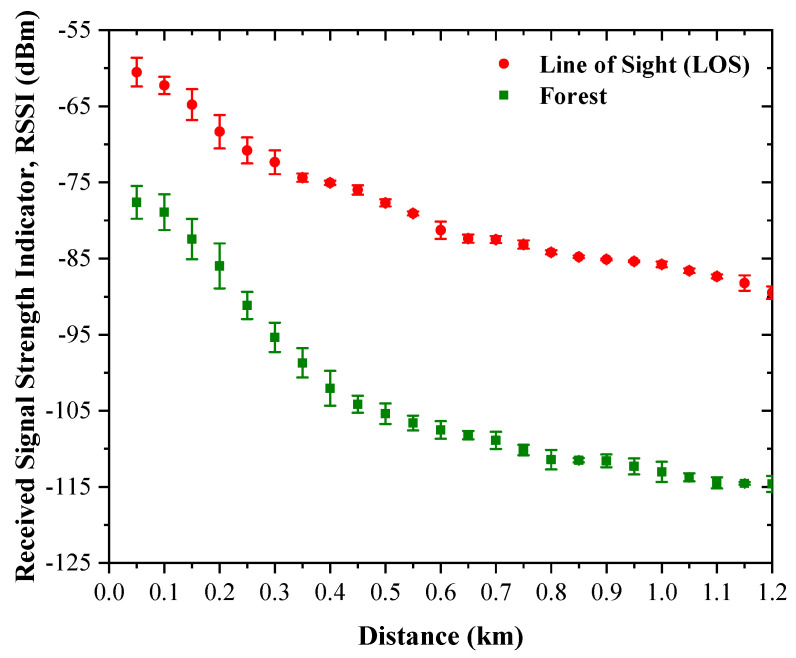
Measured RSSI as a function of transmission distance for the LOS and forested scenarios, with error bars representing the standard deviation of the repeated measurements.

The measured received signal strength indicator (RSSI) values for both the LOS and forested scenarios are presented in Figure 8 as a function of transmission distance from 50 m to 1.2 km. In both environments, the RSSI decreases progressively with increasing distance, which is consistent with the expected behavior of large-scale propagation loss. However, the rate of attenuation and the degree of measurement variability differ substantially between the two scenarios.

For the LOS scenario, the average RSSI decreases from −60.52 dBm at 50 m to −89.48 dBm at 1.2 km. The reduction is smooth and nearly monotonic throughout the measurement range. Representative values include −72.34 dBm at 300 m, −81.28 dBm at 600 m, and −85.77 dBm at 1.0 km. This gradual decay indicates that the open-path channel is dominated primarily by distance-dependent attenuation, with only limited influence from local blockage or severe shadowing.

The repeatability of the LOS measurements is also high. The standard deviation of the repeated RSSI measurements has an average value of approximately 0.78 dB and a median value of approximately 0.53 dB across all distance points. Beyond approximately 350 m, the fluctuation becomes particularly small, and from 850 m to 1.2 km the standard deviation remains mostly within 0.1–1.0 dB, with an average of approximately 0.40 dB. These results indicate that the LOS channel is stable and that the measured RSSI values are highly repeatable, making them suitable for large-scale path-loss characterization. The reported standard deviation values should be interpreted as approximate indicators of measurement variability, given the limited number of repetitions at each distance point.

In contrast, the forested scenario exhibits substantially stronger attenuation. The average RSSI decreases from −77.62 dBm at 50 m to −114.62 dBm at 1.2 km. At intermediate distances, the RSSI reaches −95.35 dBm at 300 m, −107.51 dBm at 600 m, and −113.02 dBm at 1.0 km. Compared with the LOS case, the forested link consistently shows lower received power at every observation point, demonstrating the significant influence of forest on signal propagation.

The measurement variability is also larger in the forested case. The standard deviation has an average value of approximately 1.35 dB, a median of approximately 1.15 dB, and a maximum value approaching 2.95 dB. The largest fluctuations occur in the short-to-medium range, especially from 50 m to 300 m, where the average standard deviation is approximately 2.30 dB. This behavior suggests stronger local variability caused by foliage blockage, canopy irregularity, and multipath scattering. Although the standard deviation was calculated from only three repeated measurements at each distance, it still provides a useful local indicator of link stability.

Overall, the RSSI results show that the forested environment introduces not only additional attenuation but also greater channel variability than the LOS scenario, even though both measurements were carried out under the same radio configuration and over the same distance range. This distinction is important because it confirms that forest affects both the mean received signal level and the short-range stability of the wireless link.

### 4.2. SNR Analysis and Link Quality

**Figure 9:**
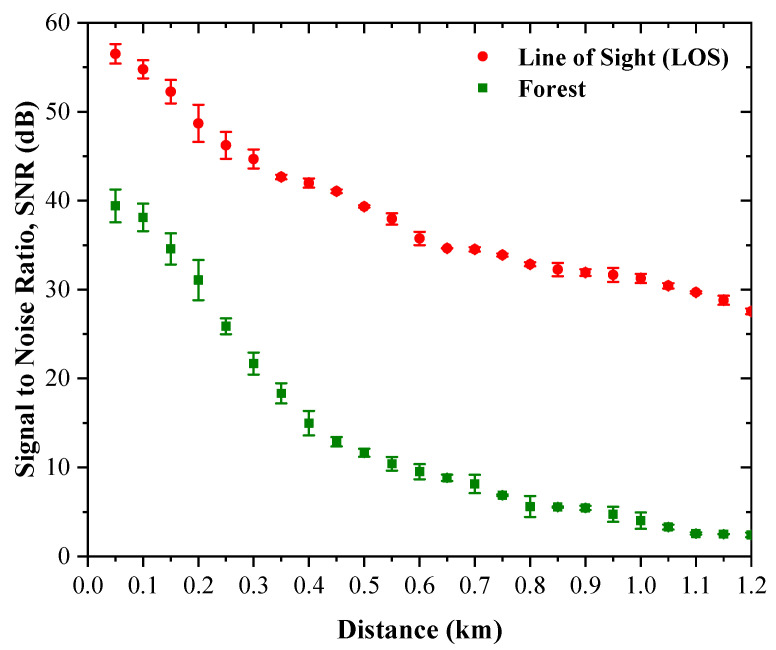
Measured SNR as a function of transmission distance for the LOS and forested scenarios, with error bars representing the standard deviation of the repeated measurements.

To complement the RSSI analysis, the communication link was further evaluated in terms of estimated signal-to-noise ratio (SNR). In this study, the SNR was not taken directly from the modem register; instead, it was calculated from the measured RSSI using the noise-floor model introduced in Section 2. Based on a receiver bandwidth of 125 kHz and an assumed receiver noise figure of 6 dB, the effective noise floor is approximately −117.03 dBm. The estimated SNR was then obtained as the difference between the measured RSSI and the assumed noise floor. Under this formulation, the estimated SNR serves as a comparative indicator of link robustness and provides an intuitive interpretation of how the measured propagation loss affects communication quality. The measured SNR values for both the LOS and forested scenarios are illustrated in Figure 9.

For the LOS scenario, the estimated SNR remains high over the full measurement range. At 50 m, the estimated SNR is 56.51 dB, indicating an exceptionally strong received signal relative to the assumed receiver noise floor. As the transmission distance increases, the SNR decreases gradually, reaching 44.69 dB at 300 m, 35.75 dB at 600 m, and 27.55 dB at the maximum distance of 1.2 km. Although the downward trend is expected due to path loss, the LOS link still maintains a substantial positive SNR at the longest distance. This result indicates that the open-path channel provides strong operating margin and stable reception under the selected radio configuration.

In the forested scenario, the estimated SNR follows the same decreasing trend but at significantly lower levels. At 50 m, the estimated SNR is 39.41 dB, which is already much lower than the corresponding LOS value. The SNR then declines rapidly with distance, reaching 21.68 dB at 300 m, 9.52 dB at 600 m, and 2.41 dB at 1.2 km. These values show that the forested environment imposes a substantial penalty on communication quality, consistent with the severe RSSI degradation reported in Section 4.1.

A direct comparison between the two propagation environments confirms the magnitude of this effect. At 1.2 km, the difference between the LOS and forested estimated SNR values is approximately 25.14 dB, which is numerically consistent with the excess attenuation identified from the RSSI results. This agreement is expected because the same assumed noise floor is used for both cases. Even so, the SNR representation is useful because it translates the measured attenuation into a link-quality metric that is easier to interpret from a communication-system perspective.

The forested results also reveal an important practical implication. Beyond approximately 800 m, the estimated SNR falls below about 6 dB, and from 1.0 km onward it decreases to roughly 4 dB or less. This indicates that the link becomes increasingly sensitive to additional channel impairment, such as foliage movement, local blockage, antenna misalignment, or temporal environmental variation. In contrast, the LOS link retains a large SNR margin over the same distance range, confirming that the open propagation environment is substantially more robust.

Overall, the estimated SNR analysis supports the conclusions drawn from the RSSI measurements. The LOS channel maintains high link quality over the full 1.2 km path, whereas the forested channel experiences a pronounced reduction in communication robustness. Therefore, in addition to increasing path loss, the forested environment also reduces the effective link margin available for reliable operation, which must be considered in practical LoRa link design for tropical monitoring deployments.

### 4.3. Comparison with the Baseline Model and Quantification of Excess Attenuation

To assess how strongly the real deployment conditions deviate from idealized propagation assumptions, the measured RSSI results were compared with the baseline free-space prediction introduced in Section 3.3. For the open 1.2 km reference link, the analytical baseline based on the free-space path loss (FSPL) model predicts a received signal level of approximately −58.34 dBm. This value represents an idealized lower-bound attenuation case and therefore provides a useful reference for evaluating the magnitude of additional loss observed in practical measurements. The excess attenuation, $A_{\mathrm{excess}}\left(d\right),$ introduced by the forested environment is defined as

(11) $$A_{\mathrm{excess}}(d)=RSSI_{\mathrm{LOS}}(d)-RSSI_{\mathrm{forest}}(d)$$

where $RSSI_{LOS}(d)$ and $RSSI_{forest}(d)$ denote the measured received signal strength at distance d for the LOS and forested scenarios, respectively. For the LOS scenario, the measured RSSI at 1.2 km is −89.48 dBm, which is approximately 31.14 dB lower than the free-space baseline. This discrepancy confirms that even in an apparently open propagation path, the received signal does not follow ideal free-space behavior. In practice, the link is affected by several additional mechanisms, including near-ground propagation, implementation losses, imperfect antenna realization, connector and feeder losses, and possible ground-reflection effects. Accordingly, the free-space model should not be interpreted as a fully accurate predictor of the real LOS link, but rather as a reference against which the practical propagation penalty can be quantified.

The deviation becomes substantially larger in the forested scenario. At the same transmission distance of 1.2 km, the measured RSSI is −114.62 dBm, corresponding to a total difference of approximately 56.28 dB relative to the free-space baseline. When the LOS deviation is subtracted from this total discrepancy, the additional environment-specific attenuation introduced by the forested path is approximately 25.14 dB. This value represents the excess loss caused by the forest-rich propagation environment under otherwise comparable transmission conditions.

**Figure 10:**
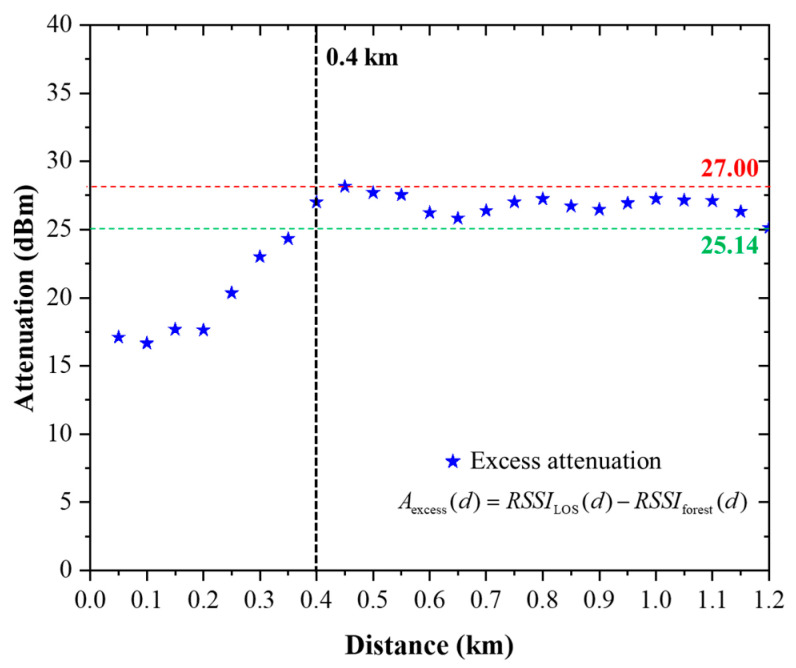
Excess attenuation of the forested scenario relative to the LOS scenario as a function of transmission distance. The results show that the forest-induced propagation penalty increases at short distances and converges to approximately 25 dB beyond 400 m.

A direct comparison between the LOS and forested measurements over the entire distance range provides additional insight into the growth of this environmental penalty, as illustrated in Figure 10. At 50 m, the forested path already exhibits an additional attenuation of approximately 17.10 dB relative to the LOS case. The excess loss increases to about 23.01 dB at 300 m, 26.23 dB at 600 m, and remains at approximately 25.14 dB at 1.2 km. Across all measurement points, the mean excess attenuation is approximately 24.70 dB. This result shows that forest-induced loss emerges at short range and remains significant over the full propagation path.

An important observation is that the forest penalty becomes relatively stable beyond the early part of the route. After roughly 350–400 m, the difference between the LOS and forested RSSI values remains mostly within the range of 25–28 dB. As clearly observed in Figure 10, the excess attenuation converges toward a nearly constant value at longer distances. This suggests that, once the propagation path is sufficiently influenced by canopy obstruction and incomplete Fresnel clearance, the forested environment behaves approximately as an additional large-scale attenuation term superimposed on the open-path loss.

Overall, these results demonstrate that a free-space-only link-budget approach is insufficient for practical LoRa deployment in forest-rich tropical environments. Instead, an environment-dependent correction term is required to account for the substantial excess attenuation observed in the forested link. This motivates the empirical interpretation developed in the next subsection.

### 4.4. Environmental Impact and Propagation Mechanisms

The comparative results presented in the previous subsections show that the forested link experiences not only lower RSSI and lower estimated SNR, but also greater measurement variability than the LOS link. These differences can be explained by the combined effects of several physical propagation mechanisms that become significant when the radio path passes through forest-rich terrain.

The first mechanism is absorption by foliage. At the operating frequency of 923.2 MHz, electromagnetic waves are attenuated as they interact with leaves, branches, and other water-containing plant structures. Although sub-GHz propagation is generally more tolerant of obstruction than higher-frequency systems, cumulative attenuation can still become substantial when the path traverses dense forest over long distances. This effect contributes directly to the lower received power observed in the forested scenario at all measurement points.

The second mechanism is scattering from irregular canopy structures. In a forested environment, the signal does not propagate through a uniform medium. Instead, branches, leaves, trunks, and gaps in the canopy create a highly nonuniform propagation channel. As a result, part of the transmitted energy is redirected away from the direct path, while other components arrive at the receiver with different delays and phases. This process increases effective path loss and also contributes to the larger standard deviation observed in the repeated forest measurements. The stronger short-to-medium-range variability seen in Section 4.1 is consistent with this interpretation.

A third mechanism is partial obstruction of the first Fresnel zone. As shown in Section 3, the estimated Fresnel zone clearance along the forested 1.2 km link is approximately 37.05%, which is well below the commonly recommended clearance condition for low-diffraction propagation. When the Fresnel zone is incompletely cleared, part of the propagating wavefront is blocked, and diffraction loss is introduced in addition to foliage attenuation. This is an important point because it indicates that the excess loss observed in the forested scenario cannot be attributed to forest absorption alone. Instead, it reflects the combined effect of foliage interaction and diffraction caused by geometric obstruction along the path.

Near-ground propagation effects may also contribute to the measured behavior in both scenarios, but their impact is more severe in the forested case because the signal interacts with a more complex environment before reaching the gateway. The transmitter height of approximately 1.7 m and the gateway height of approximately 8 m mean that the propagation path is not fully isolated from ground and environmental interaction. In the open LOS case, this primarily appears as deviation from the ideal free-space baseline. In the forested case, these effects are compounded by canopy blockage and increased multipath complexity.

Taken together, these propagation mechanisms explain the three main observations obtained from the measurements: first, the forested link exhibits substantially lower RSSI than the LOS link; second, the corresponding estimated SNR is significantly reduced, indicating smaller link margin; and third, the repeated measurements show greater local variability, reflecting the unstable nature of the obstructed propagation environment. These findings confirm that the forested path cannot be modeled adequately using free-space loss alone and that a practical LoRa design for tropical deployment should explicitly include additional environment-dependent attenuation and reduced margin.

From an engineering perspective, the results indicate that link planning in forest-rich areas should consider not only the nominal transmission distance, but also canopy density, antenna placement, and Fresnel zone clearance. Even when a communication link remains operational, as observed here, the reduction in received power and estimated SNR can substantially reduce robustness against future environmental changes. This motivates the empirical treatment developed in the next section, where the measured propagation penalty is translated into a practical correction model for deployment-oriented link design.

## 5. Empirical Modeling and Deployment Implications

### 5.1. Empirical Characterization of Distance-Dependent Propagation

To obtain a compact and measurement-based description of large-scale propagation behavior, the measured RSSI data were fitted using the standard log-distance regression model. This model is widely adopted in wireless propagation analysis because the received signal strength typically varies linearly with ${log}_{10}(d)$, where d is the transmission distance.

Accordingly, the measured RSSI is expressed as

(12) $$\begin{array}{cc} RSSI(d)=a+b{\cdot log}_{10}(d) & \end{array}$$

where a  represents the intercept and b  represents the slope obtained from regression.

The regression coefficients were determined using standard linear least-squares fitting applied to the measured RSSI data. Specifically, the transmission distances from 50 m to 1.2 km were first converted into ${log}_{10}(d)$. The corresponding measured RSSI values were then paired with these transformed distance values. A linear regression was subsequently performed to determine the best-fit coefficients a  and b  that minimize the squared error between the measured data and the fitted model.

For the LOS scenario, the resulting regression model is given by

(13) $$\begin{array}{c} RSSI_{LOS}(d)=-16.44-23.12{log}_{10}(d) \end{array}$$

where d  is expressed in meters. In this expression, the intercept −16.44  corresponds to the extrapolated RSSI value at a reference distance of 1 m, while the slope −23.12  represents the rate of signal attenuation with respect to logarithmic distance.

The slope can be directly related to the effective path-loss exponent n  through the standard log-distance formulation, where the coefficient of ${log}_{10}(d)$ is equal to −10n. Therefore,

$$\begin{array}{c} n_{LOS}=\frac{23.12}{10}\approx 2.31 \end{array}$$

This value is close to the theoretical free-space exponent (n=2) but reflects additional attenuation due to practical deployment conditions such as near-ground propagation and system losses.

For the forested scenario, the same regression procedure was applied to the measured RSSI data collected along the forest-obstructed path. The resulting empirical model is expressed as

(14) $$\begin{array}{cc} RSSI_{forest}(d)=-16.62-32.24{log}_{10}(d) & \end{array}$$

where d  is in meters. The corresponding path-loss exponent is obtained as

$$\begin{array}{c} n_{forest}=\frac{32.24}{10}\approx 3.22 \end{array}$$

This significantly higher exponent indicates that the forested environment introduces stronger attenuation compared to the LOS case, due to combined effects of foliage absorption, scattering, and partial Fresnel zone obstruction.

**Figure 11:**
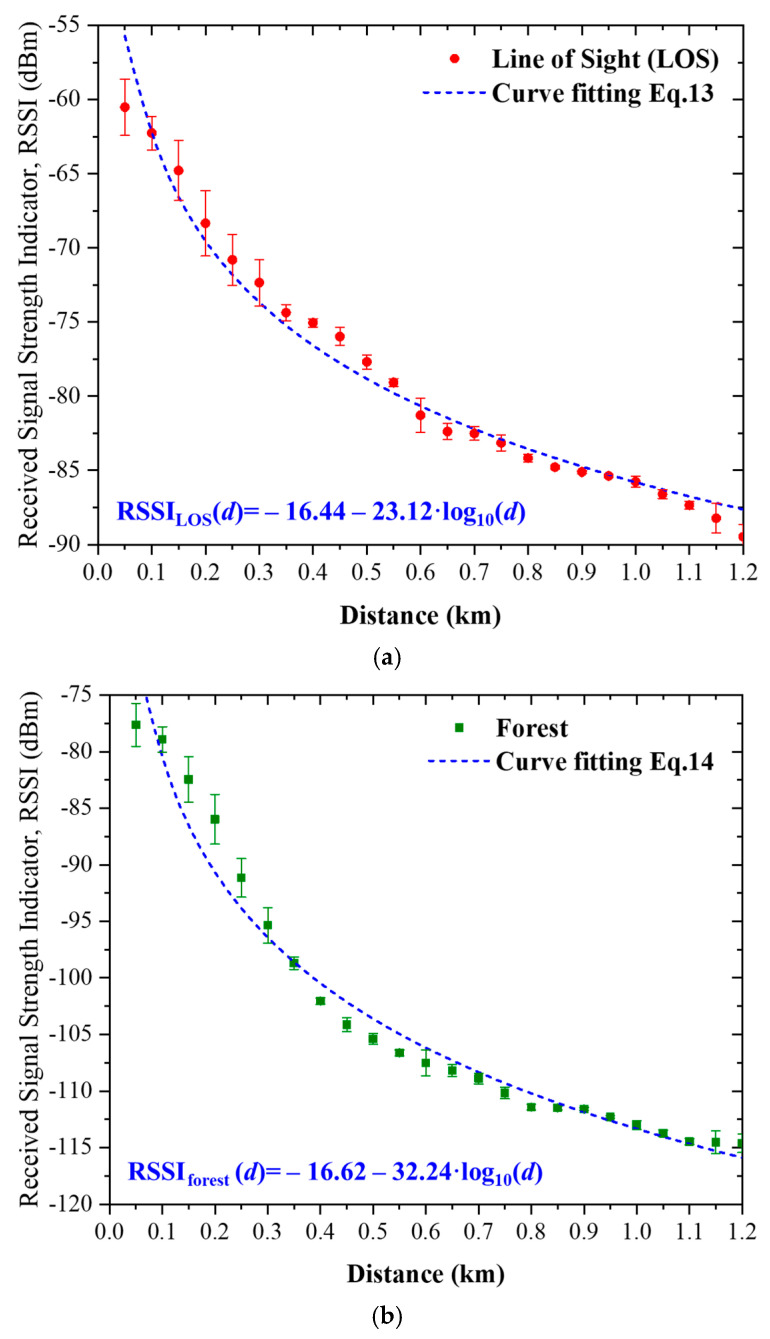
Measured RSSI data and corresponding log-distance regression fits for (**a**) LOS and (**b**) forested propagation scenarios, illustrating the difference in attenuation trends and effective path-loss behavior.

The measured RSSI data and the corresponding fitted regression curves for both propagation scenarios are illustrated in Figure 11. The fitted models provide a compact representation of the distance-dependent attenuation behavior and clearly highlight the difference between open and forest-obstructed propagation environments. In particular, the empirical models are expressed in (13) for the LOS scenario and in (14) for the forested scenario, corresponding to effective path-loss exponents of approximately 2.31 and 3.22, respectively, indicating significantly stronger attenuation in the forested environment. The coefficient of determination (R^2^) is approximately 0.97 for the LOS model and 0.96 for the forested model, indicating a strong fit. It should be noted that these empirical models are derived directly from the measured data and are therefore intended to describe the specific deployment conditions considered in this study rather than to serve as universal propagation laws.

### 5.2. Empirical Characterization of Forest-Induced Excess Attenuation

The empirical log-distance regression models for the LOS and forested scenarios, presented in Section 5.1 and illustrated in Figure 11, provide the basis for quantifying the excess attenuation between the two propagation environments. The excess attenuation introduced by the forested environment is quantified as the difference between the LOS and forested RSSI values measured at the same transmission distance, as defined in (16). At 1.2 km, the measured LOS and forested RSSI values are −89.48 dBm and −114.62 dBm, respectively, resulting in an excess attenuation of approximately 25.14 dB. Across all measured distances, the excess attenuation increases from approximately 17 dB at short range and converges to a relatively stable range of approximately 25–28 dB beyond 350–400 m, with an average value of approximately 24.70 dB, indicating saturation of forest-induced attenuation under the given propagation conditions. As clearly illustrated in Figure 10, this behavior indicates that the forested propagation path can be approximated as the LOS case with an additional large-scale attenuation term. From a practical perspective, this result supports the use of an approximate 25 dB propagation margin for preliminary link-budget design in similar forest-rich environments, while recognizing that the exact value depends on deployment geometry and environmental conditions.

For a first-order interpretation using the simplified attenuation model in Equation (8), the measured mean excess attenuation of approximately 24.70 dB over the 1.2 km forested path corresponds to an effective attenuation coefficient, α, of approximately 0.021 dB/m. This value should be interpreted only as an approximate large-scale coefficient for the specific deployment conditions considered in this study, since the measured excess attenuation includes combined effects of foliage absorption, scattering, terrain interaction, and partial Fresnel-zone obstruction.

### 5.3. Practical Implications for LoRa Link Design in Tropical Monitoring Environments

The empirical results obtained in this study have direct implications for the design of LoRa-based sensing systems in forest-rich rural environments. In the present measurement campaign, the open LOS link at a transmission distance of 1.2 km produced an average RSSI of −89.48 dBm, whereas the forested link at the same distance produced an average RSSI of −114.62 dBm. Using the assumed receiver noise floor, the corresponding estimated SNR values are 27.55 dB and 2.41 dB, respectively. These results indicate that the forested link remains operational under the selected configuration, but with a significantly reduced signal margin.

From a practical perspective, the most important design implication is that a link budget based solely on free-space or open-path assumptions would substantially overestimate the performance of a forested link. If the additional forest-induced attenuation is not accounted for, the available link margin may be overestimated by approximately 25 dB. This discrepancy is large enough to affect deployment reliability, long-term system stability, and robustness against environmental variation.

The measurements were conducted under typical hot outdoor conditions in Thailand, with ambient temperature in the range of 33–38 °C and relative humidity generally within the range of 65–71%. These values represent typical environmental conditions during the measurement period and were not directly recorded. Environmental parameters such as temperature and humidity were not explicitly monitored in this study. Future measurement campaigns will incorporate synchronized environmental sensing, including timestamped temperature and humidity logging at both the end node and gateway locations, to enable correlation analysis between environmental conditions and RSSI/SNR variations.

The measured results suggest three key design recommendations for LoRa deployment in similar tropical monitoring scenarios. First, an additional propagation margin of approximately 25 dB should be incorporated into the link budget when the propagation path includes dense forest and partial Fresnel-zone obstruction. Second, improving antenna placement and increasing Fresnel-zone clearance can be as effective as increasing transmit power, since diffraction loss and canopy interaction both contribute significantly to the observed attenuation. Third, where permitted by regulatory and system constraints, more robust communication configurations—such as higher spreading factors or improved antenna gain—should be considered when long forested links are unavoidable.

It is important to emphasize that the proposed empirical correction is site- and geometry-dependent. The present model was derived for a 923.2 MHz LoRa link in a tropical environment with antenna heights of approximately 1.7 m at the end node and 8 m at the gateway, under conditions of partial canopy obstruction and incomplete Fresnel-zone clearance. Therefore, the proposed correction should be interpreted as a practical first-order design guideline for similar deployment scenarios rather than a universal forest-loss model. Nevertheless, the results clearly demonstrate that incorporating measurement-based correction factors can significantly improve the realism and reliability of link-budget design for environmental monitoring applications.

In contrast to forested environments, urban propagation is dominated by different physical mechanisms, including strong multipath reflections, diffraction around buildings, and waveguiding effects in street canyon geometries. These mechanisms lead to different path-loss characteristics and channel variability compared to vegetation-dominated channels. As a result, urban propagation modeling requires a separate measurement and analysis framework. The present work focuses specifically on forest-rich environments, and extending the proposed methodology to urban scenarios will be considered in future studies. Future investigations will also extend the proposed measurement framework to multiple sub-GHz frequencies, including 868 MHz, 915 MHz, and 923 MHz, to evaluate the frequency dependence of vegetation-induced attenuation and propagation variability.

It should be emphasized that the empirical propagation model and the associated excess attenuation identified in this study are inherently dependent on the specific environmental conditions under which the measurements were conducted. Factors such as vegetation density, canopy structure, moisture content, seasonal variation, and terrain characteristics can significantly influence signal attenuation in forested environments. Therefore, the proposed model should be interpreted as a site-specific and condition-dependent representation, rather than a universal propagation model.

Nevertheless, the methodology adopted in this work—combining controlled field measurements, baseline comparison using the free-space path loss (FSPL) model, and extraction of environment-induced excess attenuation—can be readily extended to other deployment scenarios. By applying the same framework, location-specific propagation models and correction factors can be derived for different environments, including other forest types or climatic conditions.

From an engineering perspective, the reported excess attenuation of approximately 25 dB should be interpreted as a practical first-order design reference for environments with similar characteristics. In different forest conditions, this value may vary; however, incorporating an environment-dependent correction term remains essential for realistic link-budget design.

### 5.4. Study Limitations and Future Work

The results presented in this work should be interpreted as a controlled, site-specific measurement study conducted under fixed deployment geometry and environmental conditions. Although repeated measurements were performed at each distance point to verify measurement consistency, only three repetitions were collected per location. Therefore, the dataset is intended primarily to characterize large-scale propagation trends rather than to provide statistically exhaustive propagation modeling. The reported standard deviation values should consequently be interpreted as approximate indicators of local measurement variability under the specific conditions considered in this study.

In addition, synchronized environmental sensing, GPS-referenced logging, and detailed timestamp recording were not incorporated into the present measurement campaign. Environmental parameters such as temperature, humidity, vegetation moisture, and temporal environmental dynamics were therefore not explicitly correlated with the measured RSSI and SNR results. Furthermore, the measurements were conducted within a limited temporal window and under similar daytime environmental conditions in order to minimize uncontrolled variability during the comparative analysis.

The empirical propagation models and the corresponding excess attenuation identified in this work are inherently dependent on the specific deployment configuration, including antenna height, vegetation density, terrain characteristics, canopy structure, and Fresnel-zone obstruction conditions. Consequently, the observed excess attenuation of approximately 25 dB should be interpreted as a practical first-order engineering guideline applicable primarily to tropical forest environments with propagation conditions similar to those investigated in this study, rather than as a universal forest-loss model.

Future work will extend the proposed measurement framework to include broader spatial sampling, synchronized environmental monitoring, GPS- and time-stamped measurements, seasonal variation analysis, and multiple antenna configurations. Additional investigations across different vegetation densities, terrain profiles, operating frequencies, and environmental conditions will also be conducted to further generalize the empirical propagation model and improve the statistical robustness of the measurement dataset.

## 6. Conclusions

This study presented a comparative experimental investigation of LoRa propagation performance in two tropical rural deployment scenarios at 923.2 MHz: an open line-of-sight (LOS) link and a forested/vegetated link, evaluated under identical system configurations and a maximum transmission distance of 1.2 km. The results showed a clear difference between the two environments. The LOS link exhibited stable attenuation with RSSI decreasing from −60.52 dBm to −89.48 dBm, while the forested link experienced significantly stronger degradation, with RSSI decreasing from −77.62 dBm to −114.62 dBm and higher variability. At 1.2 km, the estimated SNR was 27.55 dB for the LOS case and 2.41 dB for the forested case, indicating that the forested link remains operational but with reduced margin. Comparison with the free-space baseline revealed substantial deviations under practical deployment conditions. The LOS and forested links showed additional attenuation of approximately 31.14 dB and 56.28 dB, respectively, corresponding to a forest-induced excess loss of approximately 25.14 dB, with an average value of about 24.70 dB. Regression analysis further yielded effective path-loss exponents of approximately 2.31 for the LOS case and 3.22 for the forested case.

The results demonstrate that free-space-based link-budget estimation alone is insufficient for practical LoRa deployment in forest-rich tropical environments. For the specific deployment conditions investigated in this study, an additional propagation margin of approximately 25 dB provides a practical first-order engineering guideline for preliminary link-budget planning. However, the exact excess attenuation is expected to vary depending on vegetation density, antenna height, terrain profile, canopy structure, and Fresnel-zone obstruction conditions.

Temporal environmental variations, including day–night cycles, vegetation dynamics (e.g., nyctinastic effects), and seasonal changes, were not explicitly considered in this study. In addition, synchronized environmental sensing, GPS-referenced logging, and statistically exhaustive sampling were beyond the scope of the present measurement campaign. Future work will extend the measurement framework to include time-dependent effects, broader spatial sampling, and environmental monitoring for more comprehensive propagation characterization.

Overall, the results of this work should be interpreted as a controlled, measurement-driven, site-specific reference for large-scale propagation behavior in tropical environments rather than as a universal or statistically exhaustive propagation model. Nevertheless, the proposed methodology and empirical observations provide useful deployment-oriented insight for LoRa-based wireless sensor network design in vegetation-rich environments.

## Figures and Tables

**Table 1:** LoRa radio configuration and measurement parameters used in the LOS and forested experiments.

Parameter	Value
Carrier frequency	923.2 MHz
Bandwidth	125 kHz
Spreading factor	SF10
Modulation	LoRa (chirp spread spectrum)
Transmitter antenna height	1.7 m
Receiver antenna height	8 m
Distance sampling interval	50 m
Number of measurement points	24
Measurements per point	3 repetitions
Receiver noise figure	6 dB
Calculated noise floor	−117.03 dBm

## Data Availability

The data presented in this study are available on request from the corresponding author.

## References

[1] Mattar C., Abdo J.B., Demerjian J., Makhoul A. (2025). Network Diffusion Algorithms and Simulators in IoT and Space IoT: A Systematic Review. J. Sens. Actuator Netw..

[2] Dauda A., Flauzac O., Nolot F. (2024). A Survey on IoT Application Architectures. Sensors.

[3] Singh R.K., Puluckul P.P., Berkvens R., Weyn M. (2020). Energy Consumption Analysis of LPWAN Technologies and Lifetime Estimation for IoT Application. Sensors.

[4] Dell’Isola M., Ficco G., Canale L., Palella B.I., Puglisi G. (2019). An IoT Integrated Tool to Enhance User Awareness on Energy Consumption in Residential Buildings. Atmosphere.

[5] Mavrogiorgou A., Kiourtis A., Perakis K., Pitsios S., Kyriazis D. (2019). IoT in Healthcare: Achieving Interoperability of High-Quality Data Acquired by IoT Medical Devices. Sensors.

[6] Łeska S., Furtak J. (2025). Procedures for Building a Secure Environment in IoT Networks Using the LoRa Interface. Sensors.

[7] Ahmed A.A. (2022). An Actor-Based Formal Model and Runtime Environment for Resource-Bounded IoT Services. Algorithms.

[8] Ullo S.L., Sinha G.R. (2020). Advances in Smart Environment Monitoring Systems Using IoT and Sensors. Sensors.

[9] Hussain R., Alhuwaimel S.I., Algarni A.M., Aljaloud K., Hussain N. (2022). A Compact Sub-GHz Wide Tunable Antenna Design for IoT Applications. Electronics.

[10] Di Serio A., Buckley J., Barton J., Newberry R., Rodencal M., Dunlop G., O’Flynn B. (2018). Potential of Sub-GHz Wireless for Future IoT Wearables and Design of Compact 915 MHz Antenna. Sensors.

[11] Gu J., Park M., Lee S., Kang H., Kim B. (2023). Evaluation of a Multi-Hop Wireless Internet-of-Things Network on Large Ships. J. Mar. Sci. Eng..

[12] Sheriff N., Kamal S., Chattha H.T., Geok T.K., Khawaja B.A. (2022). Compact Wideband Four-Port MIMO Antenna for Sub-6 GHz and Internet of Things Applications. Micromachines.

[13] Senoo E.E.K., Akansah E., Mendonça I., Aritsugi M. (2023). Monitoring and Control Framework for IoT, Implemented for Smart Agriculture. Sensors.

[14] Zhang Y., Chen J., Zeng X., Chen R., Chen L., Xiao S., Song Y. (2026). A Multimodal UAV-IoT Sensing Framework for Intelligent Pest Density Estimation in Smart Agricultural Systems. Sensors.

[15] Bagha H., Yavari A., Georgakopoulos D. (2022). Hybrid Sensing Platform for IoT-Based Precision Agriculture. Future Internet.

[16] Pham C., Ehsan M. (2021). Dense Deployment of LoRa Networks: Expectations and Limits of Channel Activity Detection and Capture Effect for Radio Channel Access. Sensors.

[17] Kumar P.P., Paul P.S., Ananda M. (2023). Development of LoRa Communication System for Effective Transmission of Data from Underground Coal Mines. Processes.

[18] González-Palacio M., Tobón-Vallejo D., Sepúlveda-Cano L.M., Rúa S., Pau G., Le L.B. (2023). LoRaWAN Path Loss Measurements in an Urban Scenario including Environmental Effects. Data.

[19] Branch P. (2022). Measurements and Models of 915 MHz LoRa Radio Propagation in an Underground Gold Mine. Sensors.

[20] Akhter F., Khadivizand S., Siddiquei H.R., Alahi M.E.E., Mukhopadhyay S. (2019). IoT Enabled Intelligent Sensor Node for Smart City: Pedestrian Counting and Ambient Monitoring. Sensors.

[21] Sangpet P., Chudpooti N., Akkaraekthalin P. (2025). Additive Manufacturing of a Passive Beam-Steering Antenna System Using a 3D-Printed Hemispherical Lens at 10 GHz. Electronics.

[22] Phakaew T., Oo T.P., Uzair M., Kowitwarangkul P., Chuchuay P., Yeetsorn R., Torrungrueng D., Chudpooti N., Chalermwisutkul S. (2025). Additively Manufactured Mechanically Tunable Cavity Resonator for Broadband Characterization of Liquid Permittivity. Sensors.

[23] Rappaport T.S. (2002). Wireless Communications: Principles and Practice.

[24] Balanis C.A. (2016). Antenna Theory: Analysis and Design.

[25] Tse D., Viswanath P. (2005). Fundamentals of Wireless Communication.

[26] Barclay L.W. (2003). Propagation of Radiowaves.

[27] Sizun H., de Fornel P., Bousquet M. (2005). Radio Wave Propagation for Telecommunication Applications.

[28] International Telecommunication Union Radiocommunication Sector (ITU-R) (2021). Attenuation in Vegetation.

[29] Ma Y., Li W., Han D., He Y., Li Q., Bai X., Xu D. (2024). Wireless Wave Attenuation in Forests: An Overview of Models. Forests.

[30] Azevedo J.A., Mendonça F. (2024). A Critical Review of the Propagation Models Employed in LoRa Systems. Sensors.

